# Alteration of Thyroid-Related Hormones within Normal Ranges and Early Functional Outcomes in Patients with Acute Ischemic Stroke

**DOI:** 10.1155/2016/3470490

**Published:** 2016-06-08

**Authors:** Xiao-yan Xu, Wen-yu Li, Xing-yue Hu

**Affiliations:** Department of Neurology, The Sir Run Run Shaw Hospital, Zhejiang University School of Medicine, Hangzhou, Zhejiang 310016, China

## Abstract

This study evaluated the prognostic value of thyroid-related hormones within normal ranges after acute ischemic stroke. This was a retrospective study and we reviewed 1072 ischemic stroke patients consecutively admitted within 72 h after symptom onset. Total triiodothyronine (T3), total thyroxine (T4), free T3, free T4, and thyroid-stimulating hormone (TSH) were assessed to determine their values for predicting functional outcome at the first follow-up clinic visits, which usually occurred 2 to 4 weeks after discharge from the hospital. 722 patients were finally included. On univariate analysis, poor functional outcome was associated with presence of atrial fibrillation as the index event. Furthermore, score of National Institutes of Health Stroke Scale (NIHSS), total T4, free T4, and C-reactive protein at admission were significantly higher in patients with poor functional outcome, whereas free T3 and total T3 were significantly lower. On multiple logistic regression analysis, lower total T3 concentrations remained independently associated with poor functional outcome [odds ratio (OR), 0.10; 95% confidence interval (CI), 0.01–0.84; *P* = 0.035]. The only other variables independently associated with poor functional outcome were NIHSS scores. In sum, lower total T3 concentrations that were within the normal ranges were independently associated with poor short-term outcomes.

## 1. Introduction

Post-stroke changes in levels of thyroid-related hormones have been reported [1, 2]. A reduction in serum triiodothyronine (T3) level without an elevation of thyroid-stimulating hormone (TSH) (i.e., low T3 syndrome) is a common complication in acute cerebrovascular disease setting [3] and reported to be associated with stroke severity and poor clinical outcomes [4–7]. Better survival after acute stroke was also found even when the cut-off point used was the median T3 levels in one study [4], showing that lower T3, even when it is within the normal range, may be associated with poorer prognosis. However, the association was not confirmed by other studies [8, 9]. Therefore, it is unclear if, in ischemic stroke patients, lower T3 levels especially within the normal range on hospital admission provide valuable prognostic information. A better knowledge of this issue may be useful for risk stratification of ischemic stroke patients and, therefore, a better use of healthcare resources. The objective of this retrospective study was to investigate the prognostic value of TSH, total T3, total thyroxine (T4), free T3, and free T4 within normal ranges in acute ischemic stroke patients.

## 2. Patients and Methods

### 2.1. Study Population

We reviewed all available charts of patients admitted with acute ischemic stroke at our acute stroke unit between January 2011 and December 2013. Inclusion criteria were (1) patients diagnosed with acute ischemic stroke by new focal neurological deficit with a corresponding lesion on magnetic resonance or delayed computer tomography scan, (2) patients admitted within 72 h after symptom onset, and (3) patients with thyroid function tests within 24 h of admission. Patients with known thyroid disease and biochemically defined overt thyroid disease and those using medications that affected thyroid function were excluded. To avoid any confounding effects, subjects with cancer, hematological diseases, severe renal or liver failure, and inflammatory or infectious diseases were excluded from the study.

### 2.2. Data Collection

Baseline characteristics, including demographic data, stroke risk factors such as a history of hypertension, smoking, diabetes mellitus, hyperlipidemia, transient ischemic attacks, coronary artery disease, and atrial fibrillation, routine blood count, admission glucose, and C-reactive protein (CRP) levels measured in the emergency department, and neurological deficits, were recorded. Blood samples for CRP were taken at the time of admission and analyzed by immunoturbidimetric method using Abbott Architect c16000 biochemistry analyzer (Abbott Laboratories Inc., Abbott Park, USA).

Thyroid function tests examined TSH, total T3, total T4, free T3, and free T4 levels. Serum concentrations of these hormones were measured by Chemiluminescent Microparticle Immunoassay (Architect, CO, USA). Normal ranges of thyroid hormones are 0.35–4.94 IU/L for TSH, 0.57–1.59 ng/mL for total T3, 4.87–11.72 *μ*g/dL for total T4, 1.71–3.71 pg/mL for free T3, and 0.7–1.48 ng/dL for free T4.

Stroke severity on admission was assessed using the National Institutes of Health Stroke Scale (NIHSS), and the distribution of NIHSS scores on admission was divided into 3 categories: mild, NIHSS < 8; moderate, NIHSS 8–14; and severe, ≥14 [6]. Stroke subtype was divided into 3 groups according to the vessels involved: anterior circulation group, posterior circulation group, and both anterior and posterior circulation group. Functional outcomes were evaluated by the modified Rankin Scale (mRS) at the first follow-up clinic visits, which usually occurred 2 to 4 weeks after discharge from the hospital. Patients were classified into 2 outcome groups: poor functional outcome (mRS > 2) and good functional outcome (mRS ≤ 2). All evaluations were performed by the study neurologists.

### 2.3. Data Analysis

Data were analyzed using SPSS 22.0 for Windows. Data are expressed as medians [interquartile range (IQR)] or mean ± standard deviation (SD) for quantitative variables and as numbers (percent) for qualitative variables. The chi-square and independent-sample *t*-test were used to compare respective categorical and continuous variables between different outcome groups. Spearman's rank correlation was performed to assess the association between thyroid hormone levels and stroke severity on admission. Pearson correlation was used to evaluate the association of CRP with thyroid hormone levels. At last, the variables with significant difference between different outcome groups were included in multiple logistic regression analysis and backward likelihood ratio test was used to find out the independent predictors of poor functional outcomes after stroke. All statistical tests were 2-tailed, and *P* < 0.05 indicated statistical significance.

## 3. Results

During the study period, there were 1072 ischemic stroke patients admitted to our acute stroke unit within 72 h after symptom onset. We excluded 179 patients for the following reasons: previous thyroid disease (*n* = 19), cancer (*n* = 51), hematological diseases (*n* = 13), severe renal or liver failure (*n* = 26), inflammatory or infectious diseases (*n* = 32), and no thyroid function test within 24 h of admission (*n* = 38). Among the remaining 893 patients, 171 had abnormal thyroid functional tests with at least one of the thyroid-related hormones below or above the normal ranges. A total of 722 patients (80.9%) had thyroid-related hormones within normal ranges and these patients were included in the final analysis.

The baseline characteristics of included patients are summarized in Table 1. More than half of the patients (*n* = 441; 61.1%) were male and the median age was 67 years (IQR 59–76 years). Most of the patients (*n* = 504; 69.8%) had strokes that involved the carotid artery system, 201 patients (27.8%) had involvement of the vertebral basilar system, and 17 (2.4%) had involvement of both circulation systems. Common vascular risk factors included histories of hypertension (66.3%), smoking (41.6%), hyperlipidemia (41.0%), and diabetes mellitus (24.4%).

There were negative correlations between NIHSS scores and levels of total T3 (*r* = −0.144, *P* = 0.000) and free T3 (*r* = −0.183, *P* = 0.000) and between CRP and total T3 (*r* = −0.195, *P* = 0.000) and free T3 (*r* = −0.206, *P* = 0.000). There were positive correlations between NIHSS scores and levels of total T4 (*r* = 0.087, *P* = 0.019) and free T4 (*r* = 0.080, *P* = 0.032), but not TSH (*P* = 0.581), and between CRP and free T4 concentrations (*r* = 0.184, *P* = 0.001). Besides, a negative correlation was found between CRP levels and free T3/free T4 ratios (*r* = −0.253, *P* = 0.000).

At the study's end point, a total of 637 patients (88.2%) had good functional outcome and 85 patients (11.8%) had poor functional outcome. As shown in Table 2, on univariate analysis, poor functional outcome was associated with presence of atrial fibrillation as the index event (*P* = 0.000). As shown in Table 3, on univariate analysis, NIHSS, total T4, free T4, and CRP at admission were significantly higher in patients with poor functional outcome, whereas free T3 and total T3 were significantly lower. Free T3/free T4 ratios were significantly higher in patients with good functional outcome than in patients with poor functional outcome. This finding indicates a reduction in peripheral T4 to T3 conversion in patients with poor functional outcome.

On multiple logistic regression analysis, lower total T3 concentrations remained independently associated with poor functional outcome [odds ratio (OR), 0.10; 95% confidence interval (CI), 0.01–0.84; *P* = 0.035]. In addition to total T3, the only other variables independently associated with poor functional outcome were NIHSS scores (OR = 1.66; 95% CI 1.51–1.82; *P* = 0.000).

## 4. Discussion

The main findings of this study were that, in ischemic stroke patients with thyroid-related hormones within normal ranges, lower total T3 concentration was an independent predictor of poor functional outcome. Greater clinical severity of stroke on admission was associated with lower T3 levels (both total and free) and higher T4 levels (both total and free).

Low T3 syndrome is common in critically ill patients, and it has been well demonstrated that low T3 syndrome was independently associated with greater mortality rate and worse functional outcomes across different populations of somatic patients, including patients after acute cardiac events [10], patients with respiratory failure [11], and intensive care unit patients [12], and after surgery for brain tumor [13]. Similar results were also reported in stroke patients. For example, it has been shown that ischemic stroke patients with low T3 syndrome were at increased risk for poor functional outcome at follow-up that was scheduled from 2 to 4 weeks after ischemic stroke [6]. Another study found that 1-year survival was significantly worse in stroke patients with low T3 syndrome [4]. Better survival after acute stroke was also found even when the cut-off point used was the median total T3 levels [4]. Both of the studies mentioned above investigated total T3 concentrations. Also, a study from Switzerland reported that lower total T3 concentrations were associated with poor functional outcomes and mortality at 90 days and at 1 year after ischemic stroke [7]. Our study expands on these reports and showed that lower total T3 levels that were still within the normal range on hospital admission were also related to greater clinical severity and independently associated with poor functional outcome after stroke. Our findings contribute the growing literature that total T3 concentrations can serve as a valuable prognostic marker in stroke patients.

While it is well documented that lower T3 serum concentrations are associated with poor disease outcomes, it remains largely unknown if the evaluation of serum T3 concentrations can improve currently routinely employed stroke prognostic models [3]. To the best of our knowledge, only one study has compared the predictive value of T3 concentrations with clinical stroke severity for patient outcome [7]. Besides, numerous important questions between thyroid hormone profile and stroke outcome [3] remain unclear nowadays. Therefore, considering routine evaluation of serum thyroid profile of stroke patients on admission for outcome prediction still needs further studies.

On the other hand, our study also showed the association between free T3 concentrations and stroke severity. And in univariate analyses, lower free T3 levels that were still within the normal range were related to poor functional outcome. The prognostic value of free T3 after stroke has been explored before. Ambrosius and colleagues have reported worse functional outcome at 30 days and 1 year and a greater mortality rate at 1 year in patients with free T3 concentrations in the lowest fT3 tertile versus the highest tertile [5]. However, the independent predictive role of free T3 concentrations for functional outcome after stroke was not confirmed in other studies [8, 9], in line with ours. In our study, in multivariate analyses, free T3 concentrations failed to predict poor functional outcomes independently. It seems that total T3 may play more important role than free T3 in predicting functional outcome after ischemic stroke. However, since none of the studies reviewed investigated the total and free fraction of T3 at the same time, the superiority of total T3 over free T3 in predicting functional outcomes after ischemic stroke needs further studies.

It is well known that, within a few hours after the onset of stroke, plasma T3 decreases, and the magnitude of the change is related to the stroke severity [14–17]. T4 also decreases in severely ill patients [16, 17]. However, we found that T4 levels increased in patients with higher NIHSS scores, and the T4 levels were higher in patients with poorer prognoses. This finding could be explained by the fact that most of the patients enrolled in our study suffered from mild stroke, so the stroke was not severe enough to influence the secretion of the thyroid hormones, but it could have reduced peripheral conversion of T4 to T3; as a result, the level of T3 decreased while the level of T4 increased. The decrease in peripheral conversion is also associated with disease severity.

Acute ischemic stroke triggers systemic inflammatory response, and proinflammatory cytokines were reported to mediate the development of the low T3 concentrations [8]. Studies in animal models demonstrated that proinflammatory cytokines interfere with thyroid hormone metabolism [18]. We did not have the chance to investigate IL-1, IL-6, IL-10, TNF-*α*, and other proinflammatory cytokines, but we had the chance to explore the association of CRP, another proinflammatory cytokine, with thyroid hormone concentrations. Our study showed that higher CRP levels were associated with lower T3 concentrations. Free T3/free T4 ratios were also significantly lower in patients with higher CRP levels. This finding showed that proinflammatory cytokines can impair the conversion of peripheral T4 to T3, resulting in low T3 concentrations. CRP is reported to be related to poor prognosis after stroke [8, 19, 20]. Our study also showed that elevated CRP concentrations were associated with poor functional outcome on univariate analysis. In this context, our study has demonstrated that decreased total T3 levels predict stroke outcome beyond systemic inflammatory response.

There are several limitations to our study. First, this is a retrospective study, and a proportion of stroke patients without thyroid function tests were not included in the cohort, thus subjecting our results to selection bias. Second, we lacked long-term follow-up of the clinical outcomes of patients. However, acute outcomes of ischemic stroke contribute to stroke care costs, which may be used as an indicator of quality care and are relevant for long-term outcomes [9]. Third, we had only a single baseline measurement and no dynamic monitoring of thyroid function. The major strengths of our study are the lack of similar investigations in ischemic stroke patients and the simultaneous measurements of serum levels of TSH, total T4, free T4, total T3, and free T3 on admission.

In conclusion, this study assessed the prognostic value of thyroid-related hormones within normal ranges in patients with acute ischemic stroke. We found that levels of total T3 that were lower, but which were within the normal ranges, were independently associated with functional outcome after acute ischemic stroke. Total T3 can be an important prognostic biomarker in patients with acute ischemic stroke.

## Figures and Tables

**Table 1:** Baseline characteristics of patients with acute ischemic stroke.

Clinical variables	
Age (years) (median, IQR)	67 (59–76)
Sex (male, %)	441 (61.1%)
Smoking (*n*, %)	300 (41.6%)
Hyperlipidemia (*n*, %)	296 (41.0%)
Coronary artery disease (*n*, %)	56 (7.8%)
Diabetes mellitus (*n*, %)	176 (24.4%)
Atrial fibrillation (*n*, %)	55 (7.6%)
Previous stroke (*n*, %)	84 (11.6%)
Transient ischemic attacks (*n*, %)	23 (3.2%)
History of hypertension (*n*, %)	479 (66.3%)

Blood pressure on admission (median, IQR)	
Systolic blood pressure (mmHg)	158 (140–174)
Diastolic blood pressure (mmHg)	82 (74–92)
Baseline NIHSS scores (median, IQR)	2 (1–4)
Mild severity at admission (NIHSS < 8) (*n*, %)	627 (86.8%)

Laboratory variables (median, IQR)	
TSH (IU/L)	1.6 (1.1–2.3)
Total T4 (*µ*g/dL)	6.6 (5.8–7.5)
Total T3 (ng/mL)	0.8 (0.7–1.0)
Free T4 (ng/dL)	1.1 (1.0–1.2)
Free T3 (pg/mL)	2.5 (2.3–2.8)
Initial WBC count (×10^9^/L)	6.9 (5.7–8.6)
Glucose on admission (mmol/L)	7.1 (5.8–9.4)
C-reactive protein (mg/dL)	1.9 (0.9–4.1)
Poor functional outcome (mRS > 2) (*n*, %)	85 (11.8%)

IQR, interquartile range; NIHSS, National Institutes of Health Stroke Scale; TSH, thyroid-stimulating hormone; T4, thyroxine; T3, triiodothyronine; WBC, white blood cell; mRS, modified Rankin Scale.

**Table 2:** Demographics and vascular risk factors according to outcome.

	Good functional outcome (mRS ≤ 2) (*n* = 637)	Poor functional outcome (mRS > 2) (*n* = 85)	*P*
Age (years) (median, IQR)	67 (58–76)	72 (60–80)	0.147
Sex (male, %)	396 (62.2%)	45 (52.9%)	0.101
Smoking (*n*, %)	272 (42.7%)	28 (32.9%)	0.086
Hyperlipidemia (*n*, %)	255 (40.0%)	41 (48.2%)	0.149
Coronary artery disease (*n*, %)	45 (7.1%)	11 (12.9%)	0.057
Diabetes mellitus (*n*, %)	153 (24.0%)	23 (27.1%)	0.540
Atrial fibrillation (*n*, %)	40 (6.3%)	15 (17.6%)	0.000
Previous stroke (*n*, %)	71 (11.1%)	13 (15.3%)	0.263
Transient ischemic attacks (*n*, %)	19 (3.0%)	4 (4.7%)	0.395
History of hypertension (*n*, %)	422 (66.2%)	57 (67.1%)	0.882

mRS, modified Rankin Scale; IQR, interquartile range.

**Table 3:** Admission clinical and laboratory findings according to outcome.

	Good functional outcome (mRS ≤ 2) (*n* = 637)	Poor functional outcome (mRS > 2) (*n* = 85)	*P*
Systolic blood pressure (mmHg)	157.4 ± 25.0	156.7 ± 22.0	0.813
Diastolic blood pressure (mmHg)	83.2 ± 13.8	83.8 ± 15.1	0.724
Baseline NIHSS scores (median, IQR)	2.0 (1–3)	10 (7–13)	0.000
TSH (IU/L) (median, IQR)	1.6 (1.06–2.27)	1.56 (1.06–2.12)	0.429
Total T3 (ng/mL) (median, IQR)	0.84 (0.74–0.95)	0.79 (0.63–0.89)	0.001
Total T4 (*µ*g/dL) (median, IQR)	6.48 (5.74–7.42)	7.13 (6.08–8.07)	0.001
Free T3 (pg/mL) (median, IQR)	2.54 (2.29–2.85)	2.39 (2.12–2.66)	0.000
Free T4 (ng/dL) (median, IQR)	1.08 (0.99–1.19)	1.13 (1.03–1.26)	0.001
Free T3/free T4 (median, IQR)	2.36 (2.08–2.68)	2.08 (1.85–2.37)	0.000
Initial WBC count (×10^9^/L) (median, IQR)	6.8 (5.6–8.6)	7.9 (6.3–9.3)	0.722
Glucose on admission (mmol/L) (median, IQR)	7.0 (5.8–9.3)	7.6 (6.0–11.4)	0.122
C-reactive protein (mg/dL) (median, IQR)	1.8 (0.9–4.1)	2.4 (1.0–4.7)	0.025

mRS, modified Rankin Scale; IQR, interquartile range; TSH, thyroid-stimulating hormone; T3, triiodothyronine; T4, thyroxine; WBC, white blood cell.
